# Elevating relevance of autoethnography in palliative care research

**DOI:** 10.1017/S1478951526103010

**Published:** 2026-06-18

**Authors:** Haryo Kunto Wibisono, Andra Pratama Juliyanto, Ahmad Yusroni, Muhammad Khairul Anwar, Nabila Septia Rosa, Weni Rosdiana

**Affiliations:** Department of Applied Public Administration, State University of Surabaya, Surabaya, Indonesia

Dear Editor,

We read with curiosity the current article by Neto (2026), which engages autoethnography in the context of palliative care (PC) inquiry. Their work examines 3 recurrent interactional patterns in PC, such as “The Palliative Honeymoon,” “The Cousin of France,” and “Do Everything!” Beyond the important clinical insights provided through their investigation the methodology behind the research is noteworthy to enter the conversation.

Autoethnography has been praised as a methodology from anthropological studies that highlights personal experience in particular cultural beliefs, practices, identities, and context (Khosravi 2016; Sparkes 2020). Autoethnography has long occupied an ambiguous scope in health research (Chang 2016), as a type of method simultaneously praised for its depth and questioned for its rigor. Neto (2026) demonstrated when analytic autoethnography combined with systematic reflexivity and scholarly integration, it can produce knowledge that neither surveys nor clinical audits can reach. Neto’s work renders *tacit knowledge*, culturally embedded logics that quietly govern how clinicians, patients, and families relate to one another at the end of life.

We would like to add several elaborations to this letter. First, the insider-researcher situation. These issues are often treated as a source of potential bias, but it can be transformed into an analytical advantage. As Warner (2024) has argued in the context of physician-researchers, insider positionality, when critically examined, affords an empathic sensitivity that enables researchers to explore the emic point of view while simultaneously generating accountability through continuous reflexivity. Neto et al. demonstrate this by clearly describing emotional responses together with their analysis. In the PC field, where researchers work closely with people who suffering, it is crucial to draw the boundary between emotional response and analytical thinking.

Second, the paper tackles an ever-present methodological weakness. As shown by Salzmann-Erikson (2024), autoethnographies in the fields of nursing and health have not been fully employed, particularly in PC research, where relational and emotional intricacies constitute the essence of daily practice. By situating their findings within an explicit theoretical framework, Neto (2026) moves autoethnography from evocative storytelling toward what Chang (2016) called as an analytic rigor.

Third, data patterns from Neto (2026) reflected what a good qualitative looks like in practice. The conversion from field note fragments, raw expression, emotionally charged, context-specific, to conceptualized and transferable clinical constructs, represents the kind of systematic interpretation that anthropologist Clifford Geertz describes as “thick description” (1973). In a field where evidence-based medicine often privileges randomized trials, Neto (2026) remind us that some of the most practice-relevant knowledge cannot be randomized but must be lived, reflected upon, and carefully articulated.

Indeed, their findings are congruent with current perspectives on family communication at the end of life. The existing literature has always acknowledged the significant influence of culture and role within the family in influencing the reaction of both patients and relatives to issues surrounding prognosis, treatment, and care (Hira et al. 2025; Ramsey et al. 2025). This is precisely what autoethnography can reveal; the dynamics from within the clinical encounter itself.

One of the limitations that should be considered is generalization of results across cultures. The observations conducted by the authors were done from a specific cultural perspective, such as Portuguese or Southern European. The results cannot be generalized to apply to other regions like Asia or Africa, where exist different cultural practices such as, spiritual value, ritual, family intimate, and filial duties. This concern has been aptly raised by the author, and further studies that involve the opinions of clinicians from different nations would definitely enhance the theoretical scope of PC research. As Neto observed, autoethnography does not aim at statistical generalization; rather, it aims at what qualitative researchers refer to as conceptual transferability (Drisko 2025). Such transferability will also be beneficial to the main objective of Neto’s study of offering guidance to healthcare professionals in difficult situations and enhancing care provisions.

This is an era in PC studies where the use of multiple methodologies cannot simply be condoned but made imperative. In the midst of globalization, PC research moving into more diverse cultural settings, the need for methodologies that can penetrate the non-measurable becomes crucial, like the intricate processes of human engagement in a setting of death, uncertainty, and love. Analytic autoethnography is one such methodology, as shown by Neto (2026), who invite others to follow.
